# Postoperative Compliance With Follow-Up Among Photorefractive Keratectomy Patients in Riyadh, Kingdom of Saudi Arabia

**DOI:** 10.7759/cureus.77237

**Published:** 2025-01-10

**Authors:** Alanoud A Alfaqih, Shaden D AlShammari, Noora A Juaythin, Sarah H Aljefri

**Affiliations:** 1 College of Medicine, Imam Mohammad Ibn Saud Islamic University (IMSIU), Riyadh, SAU; 2 Ophthalmology, Imam Mohammad Ibn Saud Islamic University (IMSIU), Riyadh, SAU; 3 Ophthalmology, King Khaled Eye Specialist Hospital, Riyadh, SAU

**Keywords:** adherence, anterior segment refractive surgery, compliance, follow-up, photorefractive keratectomy

## Abstract

Introduction

Refractive error is a condition that results when the eye fails to concentrate light rays from objects onto the retinal plane, resulting in blurry images. And it accounts for about 47% of all cases of vision impairment in high-income and developed countries.

Methods

This retrospective observational study was conducted among patients who underwent photorefractive keratectomy (PRK) at Imam Mohammad Ibn Saud Islamic University (IMSIU) Medical Center in Riyadh, Saudi Arabia. Data were collected from patients aged 18 years or older with a stable refractive error and normal corneal topography. Data were tabulated in Microsoft Excel (Microsoft Corp., Redmond, WA, USA) and were analyzed using IBM SPSS Statistics for Windows, Version 26 (IBM Corp., Armonk, NY, USA).

Results

A total of two hundred patients who underwent PRK were analyzed (male 44.5% {89} vs female 55.5% {111}). The most common age group was 18 to 30 years (81.5%, 163). The recommended four follow-ups post photorefractive keratectomy were recorded, and 100% (200) were in compliance with the first follow-up. In univariate analysis, increasing age and being an employee were the factors influencing compliance to follow-up. However, in multivariate regression analysis, being employed was the only significant independent predictor associated with follow-up compliance. Being not informed and busy schedules were recognized as the most important barriers to follow-up compliance.

Conclusion

Compliance with follow-up visits post-PRK was less than desired. However, employed patients may exhibit a better attitude toward follow-up visits than the rest. Hence, more efforts are required to educate patients about the importance of follow-ups, which may enhance their follow-up compliance.

## Introduction

Refractive error is a condition that results when the eye fails to concentrate light rays from objects onto the retinal plane, resulting in blurry images. Uncorrected refractive error ranks among the most prevalent contributors to visual impairment and represents a substantial factor in global blindness [1]. Refractive errors account for about 47% of all cases of vision impairment in high-income countries. The three main categories are myopia (short-sightedness), hyperopia (long-sightedness), and astigmatism (no single point of focus in the eye). The most clinically successful and secure refractive surgery procedures are photorefractive keratectomy (PRK), laser-assisted in situ keratomileusis (LASIK), and small-incision lenticule extraction (SMILE). Excimer laser surgery, including PRK and LASIK, are widely performed refractive surgery procedures globally. Numerous studies have documented patient satisfaction rates reaching as high as 95% following laser refractive surgery [2]. Despite having different techniques, all three types of laser vision correction have a generally similar high success rate [3-5]. In order to be considered suitable candidates for LASIK surgery, individuals must fulfill certain criteria. These include possessing a consistent and steady visual acuity, as well as maintaining realistic expectations regarding the potential outcomes of the procedure. It is imperative for patients to undergo a comprehensive evaluation conducted by a skilled and knowledgeable ophthalmologist to ascertain their eligibility for LASIK surgery [6]. Follow-up appointments are crucial for monitoring the progress of the patient's recovery after surgery. These appointments allow the ophthalmologist to assess the healing process, address any concerns or complications, and ensure that the patient's vision is improving as expected. Regular follow-up appointments during the first six months after surgery help to ensure a safe and satisfactory recovery for the patient and can also provide an opportunity to make any necessary adjustments to further optimize the visual outcome [6]. To optimize postoperative satisfaction, it is critical to systematically identify and analyze the multifactorial determinants of patient dissatisfaction. This involves evaluating preoperative expectations, intraoperative experiences, and postoperative outcomes, as well as considering psychosocial factors and effective communication throughout the surgical continuum. Employing validated assessment tools and methodologies can facilitate a comprehensive understanding of these variables, ultimately informing targeted interventions to enhance patient satisfaction [7-9]. In Saudi Arabia, it is recommended to have four follow-ups after the surgery one week postop, one month, three months, and six months postop.

This study aimed to determine PRK patient's profile, their follow-up visits, and their compliance with a post-procedure management plan. In order to analyze the relationship between patient's profile and their postop compliance, identifying causes and possible risk factors in order to improve compliance.

## Materials and methods

This retrospective observational study was conducted among patients who underwent photorefractive keratectomy (PRK) within the last two years at Imam Mohammad Ibn Saud Islamic University (IMSIU) Medical Center in Riyadh, Saudi Arabia. Ethical approval was obtained from the Institutional Review Board (IRB) for ethics in research on living creatures at IMSIU (HAPO-01-R-0011). Data were randomly collected from the medical records in addition to direct contact with each patient to ensure the consent of participation and to ensure the actual reasons for non-compliance; all participants were asked about their compliance to prescribed medication such as antibiotics, steroids, and lubricants, and if there were additional non-prescribed medication or eyedrops. A total of 200 patients aged 18 years or older with a stable refractive error and normal corneal topography within the last two years were included in this study. All patients underwent alcohol-assisted photorefractive keratectomy, and all surgeries were done by the same three surgeons at IMSIU Medical Center using Wavelight Allegretto Eye-Q 400Hz. Classification of refractive errors was high (more than 5), moderate (3-5), and mild (less than 3) refractive error. The data were analyzed using the statistical software IBM SPSS Statistics for Windows, Version 26 (IBM Corp., Armonk, NY, USA). Descriptive statistics were presented using numbers and percentages (%) for all categorical variables. The relationship between adherence to follow-up among the demographic and clinical characteristics of the patients was conducted using the Chi-square test. Significant results were then tested in a multivariate regression model to determine the significant independent predictors of follow-up compliance. A p-value of less than 0.05 was considered statistically significant.

## Results

This study analyzed 200 patients who underwent PRK. As seen in Table 1, 81.5% (163) were between 18 and 30 years old, with more than half being females (55.5%, 111). A vast majority of the patients were students (71%, 142). Previous use of contact lenses was 41.5% (83), and 81% (162) were spectacle dependents. Among the patients, 30.5% (61) were known to have dry eyes. All patients underwent PRK. All patients used MMC (mitomycin C) 0.2% and contact lenses post-PRK. Nearly all (99%, 198) underwent surgery in both eyes. The major types of refractive error were myopic astigmatism (95.5%, 191), with a low refractive error being the most common degree (57%, 114). In addition, all patients came for mandatory appointments one week postop to remove contact lenses and check the healing, but IMSUI Medical Center does not usually check for visual acuity on the first one-week follow-up. 

**Table 1:** Demographic and clinical characteristics of the patients (n=200)

Study variables	N (%)
Age group	
18 – 30 years	163 (81.5%)
31 – 40 years	34 (17.0%)
41 – 50 years	03 (01.5%)
Gender	
Male	89 (44.5%)
Female	111 (55.5%)
Occupation	
Student	142 (71.0%)
Employed	49 (24.5%)
Unemployed	01 (0.50%)
Housewife	08 (04.0%)
Preop evaluation	
Previous use of contact lens	83 (41.5%)
Current use of isotretinoin	04 (02.0%)
Previous use of isotretinoin	42 (21.0%)
History of allergic conjunctivitis	01 (0.50%)
No significant findings	107 (53.5%)
Spectacle dependence before the refractive surgery	
All time	162 (81.0%)
Sometimes	34 (17.0%)
Not using glass at all	04 (02.0%)
Any pathological eye problems	
Known to have dry eyes	61 (30.5%)
No	139 (69.5%)
Type of surgery	
Alcohol-assisted (photorefractive keratectomy)	200 (100%)
Mitomycin C (MMC) 0.2%	
Used	200 (100%)
Not used	0
Contact lens post-photorefractive keratectomy	
Used	200 (100%)
Not used	0
Surgery performed	
Binocular	198 (99.0%)
Monocular	02 (01.0%)
Type of refractive error	
Myopic astigmatism	191 (95.5%)
Myopia	04 (02.0%)
Myopia with presbyopia	02 (01.0%)
Hyperopic astigmatism	02 (01.0%)
compound astigmatism	01 (0.50%)
Degree of refractive error	
Low refractive error	114 (57.0%)
Moderate refractive error	58 (29.0%)
High refractive error	28 (14.0%)

Table 2 shows a total of four follow-ups toward the course of the treatment. Two hundred patients came to the first follow-up (100%, 200), 44.5% (89) of the patients adhered to the second follow-up, and only 3% (6) of the patients were required to finish the four recommended follow-ups.

**Table 2:** : Number of follow-ups (n=200)

Number of follow-ups	N (%)
1	200 (100%)
2	89 (44.5%)
3	18 (09.0%)
4	06 (03.0%)

The characteristics of 44.5% (89) of the patients who complied with the second follow-up are given in Table 3. It can be observed that 48.3% (43) of the patients visited the doctor from one to three months postoperatively. Among those who did not comply with the follow-up visit (N=111), the most common reason was "no knowledge" (45.5%, 50), followed by "busy" (27.3%, 30) and "no available appointment" (10.9%, 12). Visual acuity post operation was 20/20 (100%, 89), without remaining refractive error (100%, 89), and absence of corneal haze (89, 100%). However, 13.5% (12) still complained of severe dry eyes postoperatively. Compliance with antibiotics, steroids, and lubricants has been reported by 95.5% (85), 95.5% (85), and 94.4% (84), respectively. Only 4.5% (4) reported using non-prescribed medication. No other complications had been reported during the first follow-up.

**Table 3:** Adherence to second follow-up (n=89)

Variables	N (%)
How many days from the surgery?	
<1 month	36 (40.3%)
1 - 3 months	43 (48.3%)
>3 months	10 (11.2%)
Reason for not coming for scheduled follow-up	
Patient did not know	50 (45.5%)
Busy	30 (27.3%)
No appointment available	12 (10.9%)
Personal reason	03 (02.7%)
Other	15 (13.6%)
Visual acuity post-operation	
20/20	89 (100%)
Remaining refractive error (autorefraction/refraction result)	
Zero	89 (100%)
Presence of corneal haze	
Zero	89 (100%)
Presence of dry eyes and severity	
None	24 (27.0%)
Mild	32 (36.0%)
Moderate	21 (23.6%)
Severe	12 (13.5%)
Compliance to antibiotics	
Non-compliant	04 (04.5%)
Compliant	85 (95.5%)
Compliance to steroids	
Non-compliant	04 (04.5%)
Compliant	85 (95.5%)
Compliance to lubricant	
Non-compliant	05 (05.6%)
Compliant	84 (94.4%)
Use of non-prescribed medication	
Yes	04 (04.5%)
No	57 (64.1%)
Only additional lubricant	28 (31.5%)
Other complications	
No	89 (100%)
Yes	0

Measuring the relationship between adherence to the second follow-up according to the demographic and clinical characteristics of the patients (Table 4) found that older age (p=0.043) and employed (p=0.001) were more likely to comply with the second follow-up visit. No significant relationships were observed between adherence to the second follow-up in regards to gender, preop evaluation, spectacle dependence, any pathological eye problems, and degree of refractive error (p>0.05).

**Table 4:** Relationship between adherence to second follow-up among the demographic and clinical characteristics of the patients (n=200) * Some patients have multiple preop evaluations § P-value has been calculated using the Chi-square test ** Significant at p<0.05 level

Factor	Adherence to the second follow-up		P-value^§^
	Yes N (%) ^(n=89)^	No N (%) ^(n=111)^	
Age group			
≤30 years	67 (75.3%)	96 (86.5%)	0.043**
>30 years	22 (24.7%)	15 (13.5%)	
Gender			
Male	35 (39.3%)	54 (48.6%)	0.187
Female	54 (60.7%)	57 (51.4%)	
Occupation			
Student	52 (58.4%)	90 (81.1%)	0.001**
Employed	30 (33.7%)	19 (17.1%)	
Unemployed	07 (07.9%)	02 (01.9%)	
Preop evaluation*			
Previous use of contact lens	43 (48.3%)	40 (36.0%)	0.080
Previous use of Roaccutance	16 (18.0%)	26 (23.4%)	0.347
No significant findings	42 (47.2%)	65 (58.6%)	0.109
Spectacle dependence before the refractive surgery			
All time	73 (82.0%)	89 (80.2%)	0.741
Not all the time	16 (18.0%)	22 (19.8%)	
Any pathological eye problems			
Known to have dry eyes	32 (36.0%)	29 (26.1%)	0.134
No	57 (64.0%)	82 (73.9%)	
Degree of refractive error			
Low	47 (52.8%)	67 (60.4%)	0.284
Moderate to high	42 (47.2%)	44 (39.6%)	

Multivariate regression analysis shows that being employed came out as the only significant independent predictor of follow-up compliance, with an increase of 6.47-fold higher to comply with the required follow-up (AOR=6.470; 95% CI=1.218 - 34.384; p=0.028). No significant effect was observed between age and compliance to the second follow-up visits after adjustment to a regression model (p=0.768) (Table 5).

**Table 5:** Multivariate regression analysis to determine the significant predictor associated with compliance to the second follow-up (n=200) AOR: adjusted odds ratio; CI: confidence interval ** Significant at p<0.05 level

Factor	AOR	95% CI	P-value
Age group			
≤30 years	Ref		
>30 years	0.859	0.313 – 2.356	0.768
Occupation			
Student	Ref		
Employed	6.470	1.218 – 34.384	0.028**
Unemployed	2.163	0.403 – 11.622	0.368

## Discussion

This study examined the patients' compliance with follow-up post-PRK. The findings of this study revealed that postoperative compliance with follow-up among PRK patients was low, except for the first follow-up was 100% (200) to remove the contact lens. Nearly half (44.5%, 89) adhered to the second follow-up visit, decreased to 9% (18) during the third visit, and only 3% (6) patients had continued the four follow-ups. Consistent with our reports, López-Montemayo et al. (2016) also found a lack of adherence to follow-up visits among patients who underwent refractive surgery, from 18.5% at first and second visits, decreased to only 8 (7%) out of 108 LASIK patients on the sixth recommended follow-up visits in their study [6]. This has been concurred by the study of Rahman et al. (2023), who reported a decreasing trend of follow-ups between the first and second visits following cataract surgery [10]. In contrast, a study done by Yang et al. (2017) revealed that compliance on the second follow-up (75.9%) was higher than the first follow (60.8%), but it decreased significantly on third follow-up visits (26.9%) [11]. 

Data from our study suggest that older patients (24.7%, 22) who are currently employed (33.7%, 30)were more likely to adhere to follow-up visits. However, after adjustment to a regression model, only being employed remained significant, indicating an increase of six-fold higher (AOR=6.47) adherence to follow-up visits. In China, higher income, and remembering instruction from a doctor to follow up influenced adherence to ≥3 months follow-up; however, gender, age, travel cost, and postoperative satisfaction with vision showed no differences (p>0.05) [12]. This contradicted the findings of a study done in India [13]. Women aged below 70 years who had no insurance and had phacoemulsification rather than manual minor incision cataract surgery tended to attend follow-up visits, while patients with complications, poorer visual acuity at discharge, and reoperations were associated with non-compliance with follow-up recommendations. However, a study conducted in the United States found that missed visits were associated with age, estimated travel time of more than two hours, smoking, and postoperative complications, while comorbidities related to ocular, prior visits, and best-corrected visual acuity were associated with a lower rate of missed visits [14]. 

The most frequently mentioned barriers to follow-ups in our study were not having information about follow-ups, busy schedules, no appointments available, and personal reasons. Among Bangladeshi patients who underwent cataract surgery, factors influencing non-compliance to follow-ups include transport cost, unavailability of an attendant, loss of discharge letter, severe weakness, no complications with improved vision, unhelpful hospital staff, lack of enhanced vision, and combined factors [10]. Among Chinese patients who underwent the same ocular intervention, not receiving follow-up instructions, considered follow-ups unnecessary, and other personal reasons (i.e., lack of transportation, cost, physical limitation, etc.) were identified as key contributing factors for missed follow-ups [12]. The barriers to follow-up attendance could result in a decreased compliance rate, leading to undesired postoperative outcomes. Adherence to follow-up is necessary to achieve better postoperative outcomes. Better compliance with follow-ups can be achieved by targeted advertising, proper communication, and strategic communication targeting patients requiring follow-ups based on the physician's advice.

We suspect that satisfaction with postoperative outcomes of refractive surgery could also contribute to a decreased rate of follow-up visits. Patients who achieved normal vision postoperatively may not be required to complete or attend the recommended follow-ups. Several studies documented satisfaction with laser refractive surgery due to excellent postoperative outcomes [7,15,16]. Our study did not measure patient satisfaction with PRK, although it may be considered for future research. However, evidence suggests that the patient's characteristics at their second follow-up encompassed satisfaction. For instance, most patients in our study had normal visual acuity at the second follow-up, refractive errors were fixed, corneal haze was not present, and most of all, no complications had been reported postoperatively. 

This study is limited by its focus on a single hospital, with no proper availability of appointments and non information, which may restrict the generalizability of the findings. The unique patient demographics and practices of this institution may not reflect those of other healthcare settings. As a result, while the insights are valuable within this context, they may not be applicable to broader populations. Future research should include multiple sites to enhance the applicability of the findings across diverse environments.

## Conclusions

There was a lack of adherence to follow-up among patients who underwent PRK. Compliance with follow-up may vary significantly according to age and occupation but not with gender, spectacle dependency, and refractive error degree. Not being informed and having busy schedules are the key contributors to non-compliance to follow-up. Appropriate education could help patients achieve better follow-up attitudes. This approach could enhance attendance at follow-up visits and could definitely improve postoperative outcomes and compliance.
